# A multi-agent RAG system for generating SCORM courses from enterprise documents

**DOI:** 10.3389/frai.2026.1834985

**Published:** 2026-06-01

**Authors:** Gulshat Amirkhanova, Bauyrzhan Amirkhanov, Alikhan Amirkhanov, Ramilya Aubakirova

**Affiliations:** Department of Artificial Intelligence and Big Data, Faculty of Information Technology, Al-Farabi Kazakh National University, Almaty, Kazakhstan

**Keywords:** automated course generation, corporate onboarding, enterprise learning, retrieval-augmented generation, SCORM

## Abstract

Corporate onboarding requires the effective transfer of complex organizational knowledge embedded in internal policies and procedural documents; however, existing artificial intelligence (AI)-driven course generation systems primarily target academic or public knowledge domains. This gap limits the scalability and consistency of enterprise training, particularly in regulated environments where factual accuracy is critical. In this study, we present a multi-agent pipeline that automatically generates Sharable Content Object Reference Model (SCORM) 1.2-compliant e-learning courses from heterogeneous enterprise documents using large language models and retrieval-augmented generation (RAG). The system integrates four stages: semantic document ingestion with structure-aware chunking and embedding, an autonomous ReAct-based architect agent for course design, a parallel content generation pipeline combining multi-query retrieval and neural reranking, and standards-compliant SCORM packaging for deployment in learning management systems. Evaluated using real-world occupational safety documents, the system produced a complete multi-module course with structured lessons and assessments within minutes, demonstrating end-to-end automation of instructional design grounded exclusively in source materials. By ensuring traceability of generated content to organizational knowledge, the approach reduces the risk of hallucinations.

## 1 Introduction

Corporate onboarding represents a critical yet resource-intensive process through which organizations transfer complex institutional knowledge to new employees. Training materials are typically authored manually from internal policies, procedural guidelines, and operational documentation—a process that is time-consuming, difficult to scale, and rarely systematically updated. Research suggests that structured on-the-job training offers the strongest facilitation of organizational socialization, although evidence remains limited in certainty (Frögéli et al., 2023). Emerging approaches, including artificial intelligence (AI)-powered chatbots leveraging retrieval-augmented generation (RAG) (Frischen and Fiebig, 2025) and simulation-based tacit knowledge transfer (Dostál, 2022), show considerable promise in addressing these inefficiencies. Nevertheless, existing systems have been developed almost exclusively for academic and public knowledge contexts, leaving enterprise onboarding automation largely unaddressed.

Significant research efforts have been directed toward automating the generation of educational content. Anwar et al. (2025) presented the AI course generator (ACG), which applies machine learning and natural language processing to generate contextually relevant lesson plans, quizzes, and resource recommendations from user preferences through a modular architecture supporting domain-specific knowledge bases. Diwan et al. (2023) proposed an alternative approach that uses GPT-2 to automatically generate narrative fragments embedded into learning trajectories to improve learner engagement. Abbasi et al. (2024) described AutoLMS, which transcribes video lectures via AssemblyAI and subsequently generates summaries and assignments using OpenAI models. A practical case study demonstrated that a complete “Multimedia Databases” course of 87 pages could be generated using ChatGPT in under 1 day, achieving an originality index of 8.7–13%. Morales-Chan et al. extended this approach to Massive Open Online Course (MOOC) design by integrating ChatGPT for scripting, DALL-E 2 for visual content generation, and HeyGen for photorealistic synthetic instructor avatars. Althaf et al. (2025) developed SARA AI, a collaborative platform enabling users to generate courses from keywords while editing course structure and adding custom code examples and media. The MotorIA system distinguished itself by automatically constructing knowledge graphs and course indices from unstructured sources, including Wikipedia articles and PDF documents.

Parallel research has addressed the integration of AI-generated content with existing learning management systems (LMSs). Mzwri and Turcsányi-Szabo (2025) introduced the dynamic course content integration (DCCI) mechanism, which dynamically retrieves content from Canvas LMS to provide context-dependent responses through an “Ask ME” assistant; a pilot study with 120 programming students confirmed high satisfaction scores of 4.65 out of 5 and reduced psychological barriers to seeking help. Ioannou-Sougleridi et al. (2024) proposed MoodleSense, an architecture coordinating knowledge assessment, transformation of content into interactive simulations, and automatic translation of materials. Arghir (2024) demonstrated a Tutor LMS implementation on WordPress using OpenAI plugins to adapt theoretical materials across different difficulty levels.

Despite this breadth of research, a fundamental gap remains: No existing system addresses the generation of structured training content from proprietary organizational documents such as internal compliance policies, operational procedures, and enterprise guidelines. Corporate knowledge is heterogeneous, domain-specific, and inaccessible to general-purpose language models through pretraining. Generating training content without grounding in organizational documents risks producing hallucinated or non-compliant material—an outcome that is unacceptable in regulated enterprise environments.

We present a multi-agent pipeline that closes this gap by automatically generating SCORM 1.2-compliant e-learning courses from heterogeneous enterprise documents. The system comprises four stages: semantic document ingestion, autonomous course architecture design via a ReAct agent, parallel retrieval-augmented lesson and assessment generation with neural reranking, and direct packaging for deployment to standard LMS. By grounding all generated content exclusively in organizational source documents, the system ensures factual fidelity to internal organizational knowledge while eliminating the manual authoring bottleneck that limits corporate onboarding scalability.

### 1.1 Contribution summary

This study makes the following contributions:

- We introduce a multi-agent RAG pipeline that transforms heterogeneous enterprise documents into SCORM 1.2-compliant e-learning courses without manual instructional authoring.
- We integrate structure-aware document ingestion, autonomous ReAct-based course architecture design, multi-query retrieval, neural reranking, and SCORM packaging into a single end-to-end workflow.
- We demonstrate deployment through both a Telegram bot interface and a standards-compliant LMS, showing that generated courses can be uploaded and launched without custom LMS integration.
- We provide an intrinsic retrieval evaluation on a separate labor legislation corpus to quantify dense retrieval behavior under corpus-wide and document-scoped settings.

### 1.2 Research questions

This study aims to address the identified gap in enterprise learning automation by investigating the following research questions:

*RQ1*: To what extent can a multi-agent RAG pipeline autonomously transform heterogeneous enterprise documents into a complete, structured, and SCORM 1.2-compliant e-learning course without manual instructional authoring intervention?

*RQ2*: Does grounding content generation exclusively in proprietary organizational source documents mitigate the risk of hallucination and ensure factual fidelity sufficient for deployment in regulated corporate environments?

*RQ3*: How does dense retrieval performance vary between corpus-wide and document-scoped configurations in an enterprise regulatory corpus, and to what extent do multi-query retrieval and neural reranking compensate for cross-document disambiguation errors?

## 2 Method

### 2.1 System overview

The proposed system is a four-stage multi-agent pipeline that transforms heterogeneous enterprise documents into SCORM 1.2-compliant e-learning courses without manual authoring intervention. The pipeline accepts PDF, DOCX, and PPTX files as input and produces a deployment-ready ZIP archive compatible with standard LMSs, including Moodle, iSpring, and SAP SuccessFactors. The four sequential stages are as follows: document ingestion and indexing, autonomous course architecture design, parallel content and assessment generation, and SCORM packaging. Each stage is described in detail in the following subsections. The system is implemented in Python, is platform-independent, and exposes a command-line interface and a Telegram bot frontend for production deployments.

### 2.2 Document ingestion

The ingestion stage is responsible for converting raw organizational documents into semantically indexed chunks suitable for retrieval. The pipeline entry point scans the specified input directory at the top level for files with extensions .pdf, .docx, and .pptx using non-recursive glob matching. For each discovered file, a smart skip mechanism determines whether both a summary JSON file exists on disk and the corresponding document chunks are already stored in the vector store; files satisfying both conditions are skipped to avoid redundant reprocessing.

New files are converted to a unified document representation using Docling, with format-specific options configured for PDF, Word, and PowerPoint inputs. Optical character recognition is disabled. Each conversion is executed in a separate thread via asyncio.to_thread() to prevent blocking the event loop, with files processed sequentially.

Each converted document is processed using Docling’s HybridChunker, which preserves hierarchical document structure during segmentation—an approach supported by evidence that structure-aware chunking measurably improves RAG performance (Lu et al., 2025; Tinh and Phuong, 2025; Nguyen et al., 2025; Chen et al., 2025). Chunks are tokenized using Qwen/Qwen3-Embedding-8B with a 2048-token maximum, and each chunk is contextualized by prepending its heading hierarchy, enabling structurally informed downstream retrieval. Metadata, including document identifier, name, chunk index, and headings, is retained at the chunk level. Full chunking and retrieval hyperparameter values with justifications are reported in Supplementary material S4.

All chunks are embedded using Qwen3-Embedding-8B served via a vLLM-backed inference server on port 8,001, accessed through LangChain’s OpenAIEmbeddings interface in batches of 100. The resulting vectors and associated metadata are stored in a persistent ChromaDB collection named document_chunks using cosine similarity, in batches of 500.

In parallel with chunking and embedding, the language model generates a summary and table of contents for each new document. For documents not exceeding 100,000 tokens, a single language model call produces both artifacts. For larger documents, a map-reduce strategy is applied: Sections are summarized in parallel and then consolidated in a final reduce call. All summaries are maintained as JSON files containing the document identifier, document name, summary text, table of contents, and generation method.

### 2.3 Architect agent

The course architecture stage employs a LangGraph ReAct agent that autonomously analyzes the ingested document corpus and produces a structured course outline. The agent operates in a think-act loop, iteratively invoking tools until it produces a final JSON course structure.

The agent is initialized with a system prompt defining its role as a Course Architect. If the user specifies learning goals via the goal command-line argument, these are appended to the system prompt as a User-Defined Learning Goals section. The agent has access to four tools implemented as closures over the application settings, vector store, and the embeddings client:

1. list_documents() reads all summary JSON files from the summaries directory and returns a list of document identifiers and names.
2. get_document_summary(doc_id) returns the full summary text for a specified document.
3. get_document_toc(doc_id) returns the table of contents for a specified document.
4. search_documents(query, doc_id?) embeds the query using the embeddings client, performs a top-20 cosine similarity search in ChromaDB with optional document-level filtering, and returns the most relevant chunks, truncated to 1,000 characters each.

The agent typically begins by listing all available documents, then retrieves summaries and tables of contents, and subsequently performs targeted semantic searches to clarify content details. Based on this analysis, the agent generates a JSON object conforming to the schema: {title, description, modules: [{title, lessons: [{title, description, objectives: [text]}]}]}.

The JSON is extracted from a markdown code block or parsed as raw JSON, sanitized to remove trailing commas and comments, and validated into a CourseStructure dataclass.

The generated structure is presented to the user via the command-line interface, who may approve it, request regeneration, or edit the course title. An alternative configuration-driven mode allows users to supply a YAML file defining the course structure directly, bypassing the agent entirely. The approved structure persisted as course_structure.json in the output directory.

**Figure 1:**
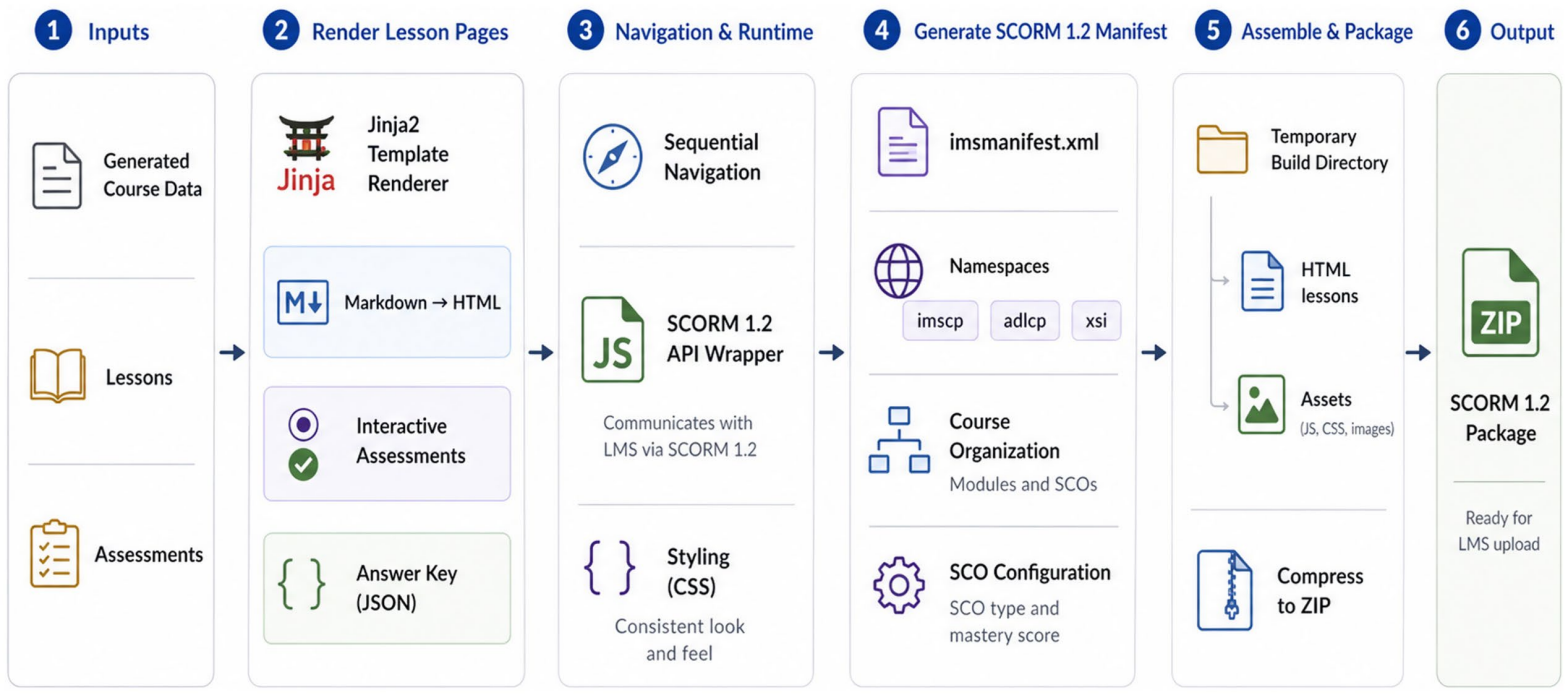
SCORM 1.2 package assembly and generation pipeline. A schematic representation of the six-step modular pipeline for converting generated course data into a deployable SCORM 1.2 package. The process encompasses input collection, lesson page rendering via a Jinja2 template engine, implementation of a SCORM 1.2 API wrapper for sequential navigation, generation of the imsmanifest.xml file, and final assembly into an LMS-ready ZIP archive.

Raw enterprise documents are parsed, chunked, embedded via Qwen3-Embedding-8B, and stored in ChromaDB alongside LLM-generated summaries, forming the retrieval knowledge base for subsequent stages (Figure 1).

**Figure 2:**
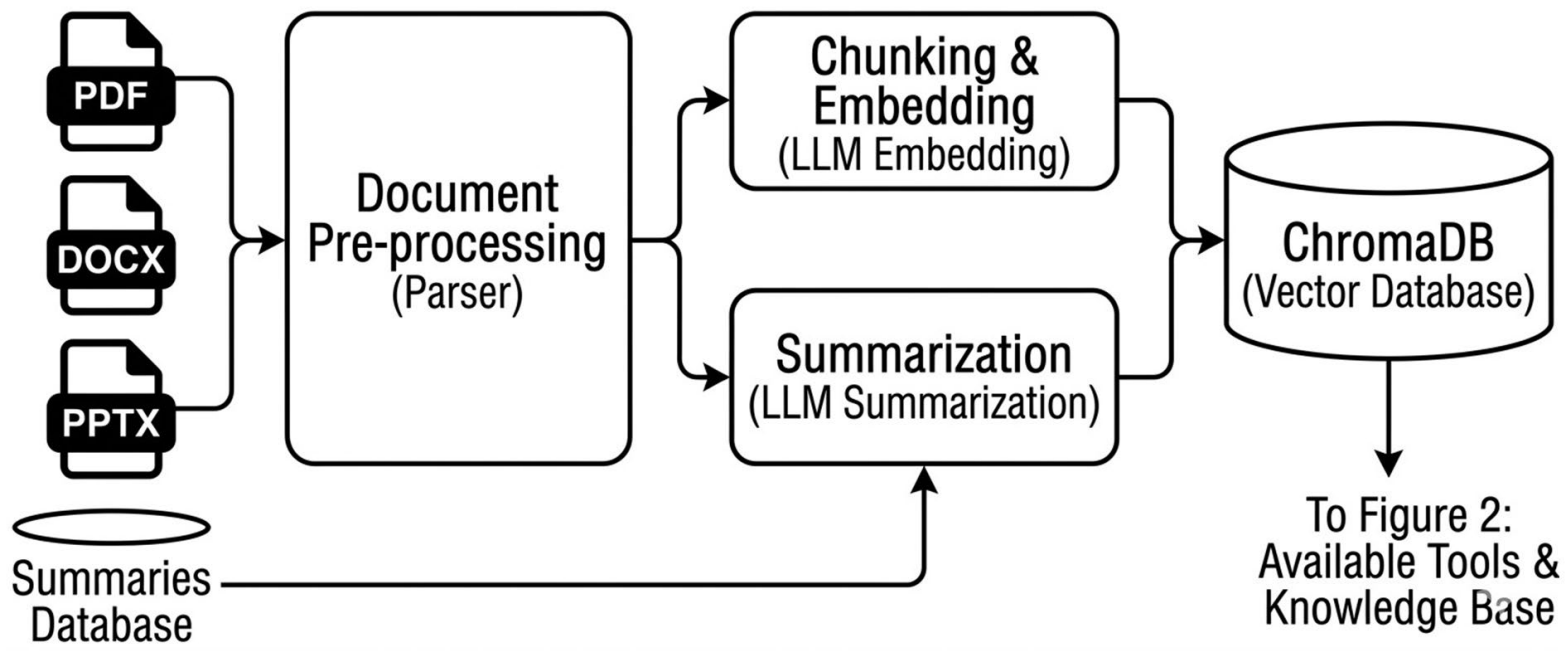
Document pre-processing and vectorization architecture. Flow diagram detailing the initial ingestion phase for raw educational materials. Standard document formats (PDF, DOCX, and PPTX) are processed through a parsing module before splitting into two parallel tracks: text chunking and LLM-based embedding into a ChromaDB vector database and an LLM-driven summarization process stores outputs in a dedicated summaries database for downstream course structuring.

A LangGraph ReAct agent iteratively queries the knowledge base to produce a modular course outline, which is subject to human review before proceeding to content generation (Figure 2).

**Figure 3:**
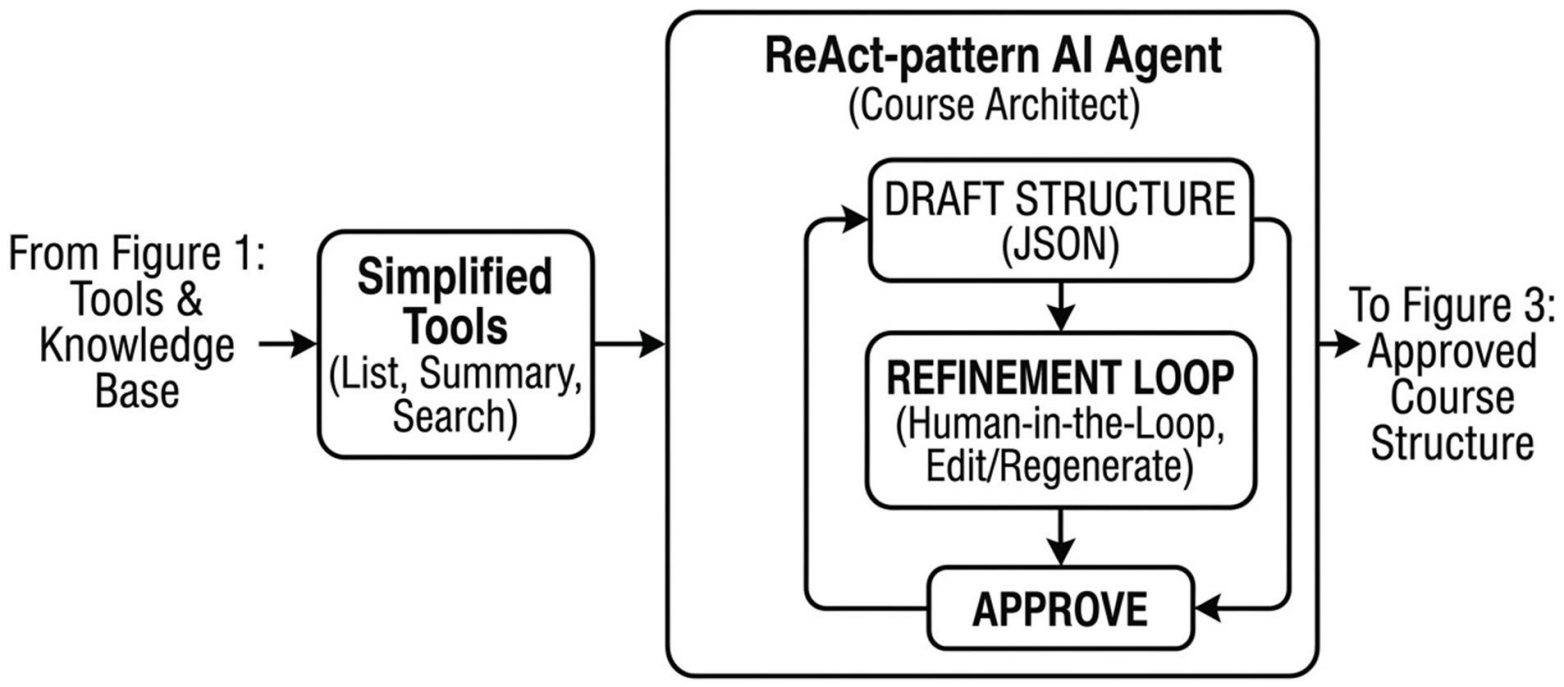
ReAct-pattern AI agent for iterative course structuring. The architectural workflow of the “Course Architect,” a ReAct-pattern AI agent. Using a set of simplified tools (List, Summary, and Search) and knowledge base inputs, the agent generates an initial draft structure in JSON format. This draft is refined through a loop incorporating human-in-the-loop feedback and edit and regenerate cycles until an approved course structure is achieved.

Each lesson undergoes multi-query retrieval, neural reranking, LLM-based content authoring, and programmatic assessment validation with automatic retry on constraint violations (Figure 3).

**Figure 4:**
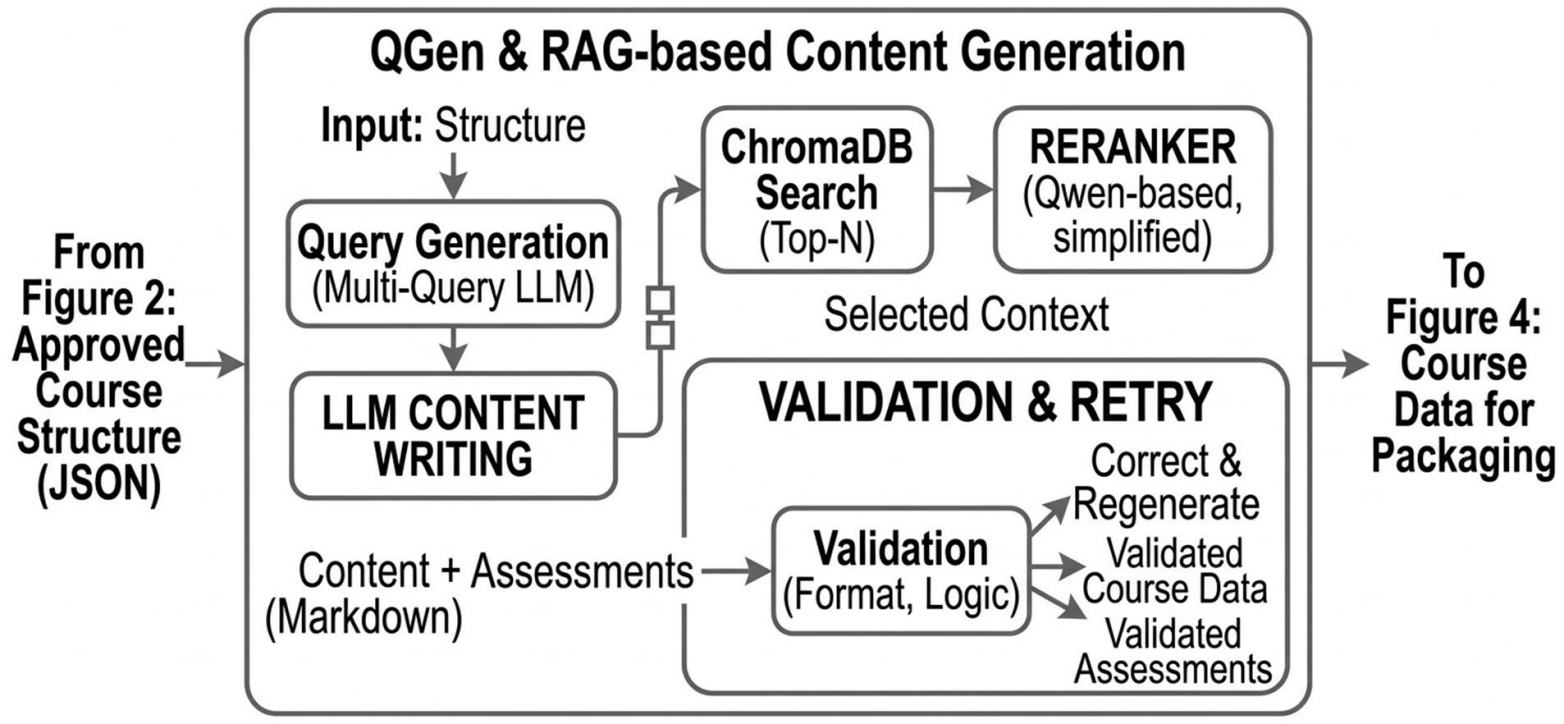
Retrieval-augmented generation (RAG) content generation workflow. This flowchart illustrates the transformation of an approved JSON course structure into raw educational content. The workflow relies on a multi-query LLM for query generation, followed by ChromaDB-based retrieval and a Qwen-based reranker to provide targeted context. The LLM-based content writing phase produces Markdown texts and assessments, which undergo strict format and logic validation with a built-in retry mechanism prior to packaging.

Validated course content and assessments are rendered to HTML via Jinja2, integrated with a generated SCORM 1.2 manifest, and compressed into a deployment-ready ZIP archive compatible with standard LMS platforms (Figure 4).

### 2.4 Content generation pipeline

Content generation is implemented as a LangGraph graph with fan-out and fan-in behavior. All lessons extracted from the approved CourseStructure are distributed in parallel via the Send API, with each lesson processed independently through a process_lesson node. Results are accumulated via an operator.add reducer and reassembled in a final assembled node.

Each lesson undergoes five sequential steps:

*Query generation*: The language model generates three to five semantically diverse search queries based on the lesson title and learning objectives. The generation function is decorated with a retry_async decorator applying exponential backoff with a maximum of three attempts. If JSON parsing of the model response fails, fallback queries derived directly from the lesson title and objectives are used.

*Multi-query retrieval*: Each generated query is embedded independently and used to retrieve the top 50 similar chunks from ChromaDB, with optional filtering by document identifier. Results across all queries are deduplicated by chunk text content, producing a set of unique candidate chunks.

*Neural reranking*: The deduplicated candidate chunks, truncated to a combined length of 24,000 characters, are submitted to Qwen3-Reranker-8B served via vLLM on port 8,002 using the /score endpoint. The reranker scores each chunk against the lesson title as the query and returns the top 20 chunks ranked by relevance score.

*Content generation*: The top 20 reranked chunks are provided to the language model as numbered sources formatted as [Source 1], [Source 2], and so on, with an instruction to generate lesson content exclusively from the provided sources. The generation is protected by a three-attempt retry decorator. The output is Markdown-formatted lesson content accompanied by truncated source chunk references for citation purposes.

*Assessment generation and validation*: The language model receives the lesson content truncated to 8,000 characters along with source chunks and generates a structured assessment in JSON format comprising three to five multiple-choice questions, each with four options and exactly one correct answer, two to three true-or-false statements, and one matching exercise with four to six pairs. The generated assessment is validated programmatically: Multiple-choice questions must have exactly one correct answer, and matching exercises must have no duplicate entries in either left or right columns. If validation fails, a retry prompt describing the specific errors is submitted for up to one additional generation attempt. If JSON parsing fails entirely, an empty assessment object is returned with a warning.

*Configuration assumptions and scalability boundaries*: The reported configuration is intended for small- to medium-sized enterprise training corpora consisting primarily of textual PDF, DOCX, and PPTX documents with a clear procedural or regulatory structure. The 2,048-token chunking limit and heading-aware contextualization are suitable for policy documents, safety instructions, onboarding manuals, and procedural guidelines in which relevant information is usually localized within sections or subsections. The 24,000-character re-ranker window is expected to be adequate when the deduplicated candidate pool for a lesson remains compact enough for the most relevant passages to be retained for cross-encoder scoring and subsequent generation. Justifications for all retrieval and generation hyperparameters are provided in Supplementary material S4; all system prompts are provided in Supplementary material S3.

The configuration may become less reliable for substantially larger repositories, highly redundant cross-document collections, scanned PDFs requiring OCR, documents dominated by large numerical tables, and manuals where answers depend on long-range cross-references across many sections. In such cases, the re-ranker window can truncate potentially relevant candidate passages, while fixed-size chunking may split tables, exceptions, or multi-step procedures across retrieval units. Scaling to multi-hundred-document repositories would require additional retrieval controls, such as document-level routing, hierarchical retrieval, metadata filtering, table-aware chunking, staged reranking, caching, and resource-aware orchestration.

### 2.5 SCORM packaging

The packaging stage converts the generated course content and assessments into a SCORM 1.2-compliant ZIP archive. A Jinja2 renderer processes a lesson HTML template for each lesson, converting Markdown content to HTML using the python-markdown library with tables, fenced_code, codehilite, and toc extensions enabled. Interactive assessments are embedded directly in each lesson page: Multiple-choice questions use radio button inputs, true-or-false items are presented as binary selections, and matching exercises present shuffled right-column options. All correct answers are serialized as JSON and passed to client-side JavaScript for in-browser response validation. Sequential navigation between lessons is provided through previous and next controls.

A SCORM 1.2 manifest file, imsmanifest.xml, is generated with proper namespace declarations for imscp, adlcp, and xsi schemas. The manifest defines the course organization as a hierarchy of modules and lessons represented as items, with each lesson declared as a Sharable Content Object with adlcp:scormtype set to sco. A mastery score threshold is embedded in each SCO via adlcp:masteryscore. Shared static assets, including a JavaScript SCORM Runtime Environment API wrapper for communicating progress and scores to the host LMS and a CSS stylesheet, are packaged as a separate asset resource.

All files are assembled in a temporary directory and compressed into a ZIP archive using DEFLATE compression. The archive filename is derived from the sanitized course title. The final archive is written to the specified output directory and is ready for upload to any SCORM 1.2-compatible LMS.

### 2.6 Telegram bot Interface

The system additionally exposes a Telegram bot frontend as an alternative interface for production deployments. The bot implements finite state machine states corresponding to the four pipeline stages: upload, architect, generate, and complete. A SQLite-backed job queue manages concurrent pipeline executions, with a job tracker providing real-time progress updates to users. An administrative panel supports operational monitoring. The bot orchestrates the same underlying pipeline as the command-line interface, providing equivalent functionality through a conversational interface accessible from mobile devices.

## 3 Results

The evaluation focuses on system behavior, structural-level generation quality, and retrieval performance. No human learning outcomes are measured in this study; all reported metrics concern pipeline execution, course structure, SCORM compliance, and information retrieval (IR) performance.

### 3.1 End-to-end pipeline behavior on the GOLD SAPA corpus

**Figure 5:**
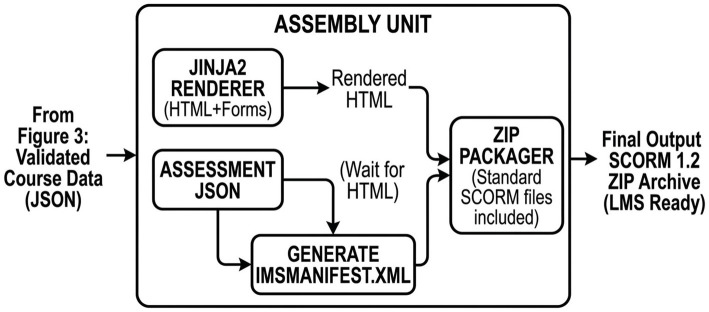
Assembly unit architecture for final SCORM packaging. A detailed schematic of the Assembly Unit responsible for finalizing the course data. A Jinja2 renderer generates HTML and forms components from the validated JSON course data. These elements are synchronized with the assessment JSON to automatically construct the imsmanifest.xml file. Finally, a ZIP packager combines all SCORM standard files and rendered HTML into a single, LMS-ready archive.

**Figure 6:**
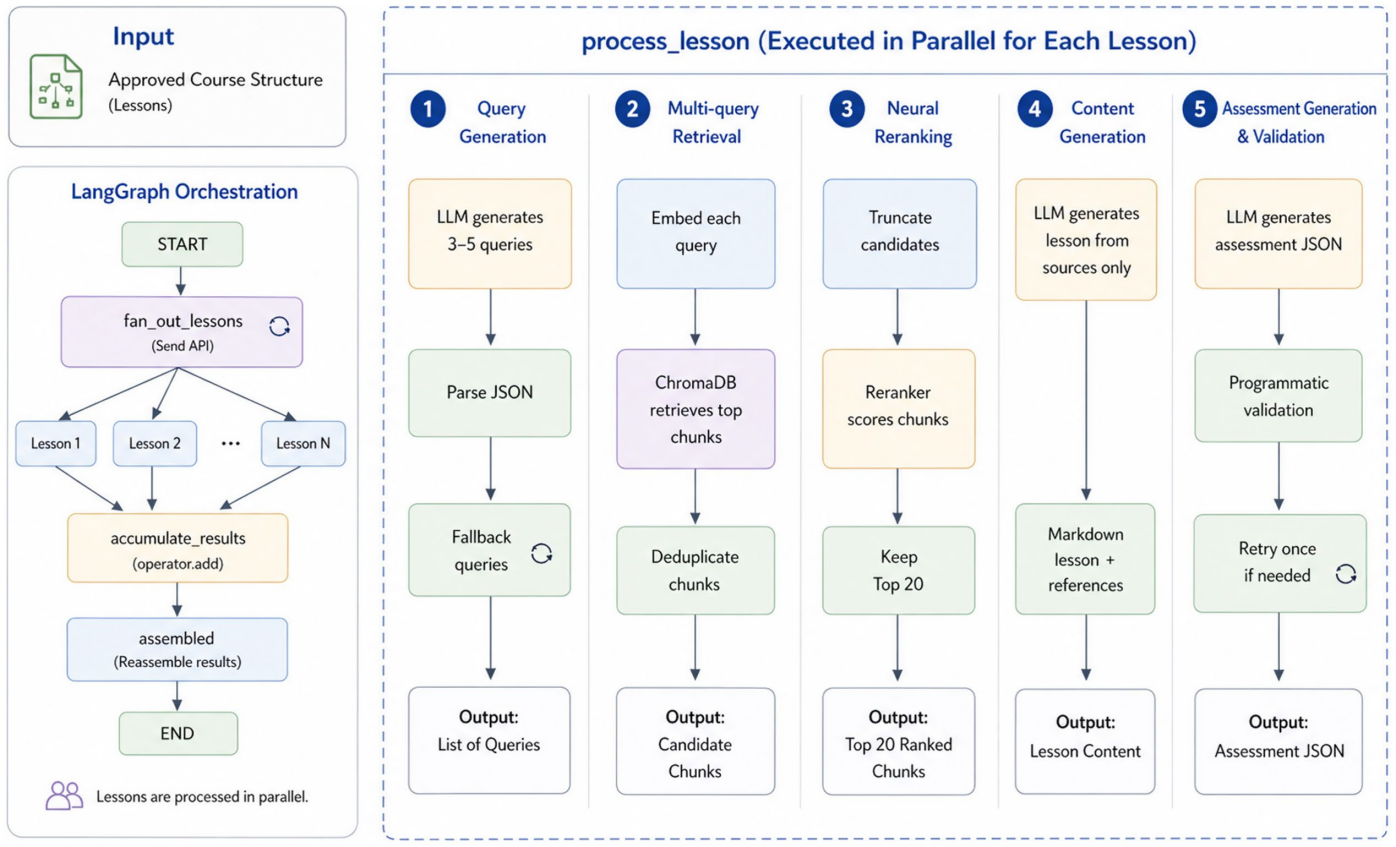
LangGraph orchestration for parallel lesson processing. The schematic displays the parallel execution architecture used to process multiple lessons simultaneously. Managed by LangGraph orchestration, the process_lesson function executes a five-stage sequence for each lesson concurrently: multi-query generation with fallback loops, neural retrieval via ChromaDB, candidate reranking to the top 20 chunks, LLM-based content generation formatted in Markdown, and programmatic assessment generation with automated validation and retries.

The system was evaluated on a corpus of three DOCX-format occupational safety documents from a bread manufacturing enterprise (GOLD SAPA Bakery), totaling approximately 1,000 words across 152 paragraphs. The corpus comprised an occupational safety instruction for bakers, a general onboarding safety briefing, and a fire safety procedure—representing the heterogeneous regulatory documentation typical of small manufacturing enterprises. Documents were submitted via the Telegram bot interface without specifying explicit learning goals, allowing the Architect agent to derive course structure autonomously. The ingestion stage was completed in 1 min 45 s; the complete SCORM-compliant ZIP archive was produced within 16 min of initial upload (Figures 5, 6).

The Architect agent autonomously decomposed the three-document corpus into a course comprising 4 modules and 16 lessons (4 lessons per module), without any user-specified learning goals. The generated modules covered general occupational safety provisions, safe operation of production equipment, fire safety and emergency response procedures, and sanitation-specific requirements for production facility staff—directly reflecting the thematic coverage of the source documents.

### 3.2 LMS deployment and interaction characteristics

**Figure 7:**
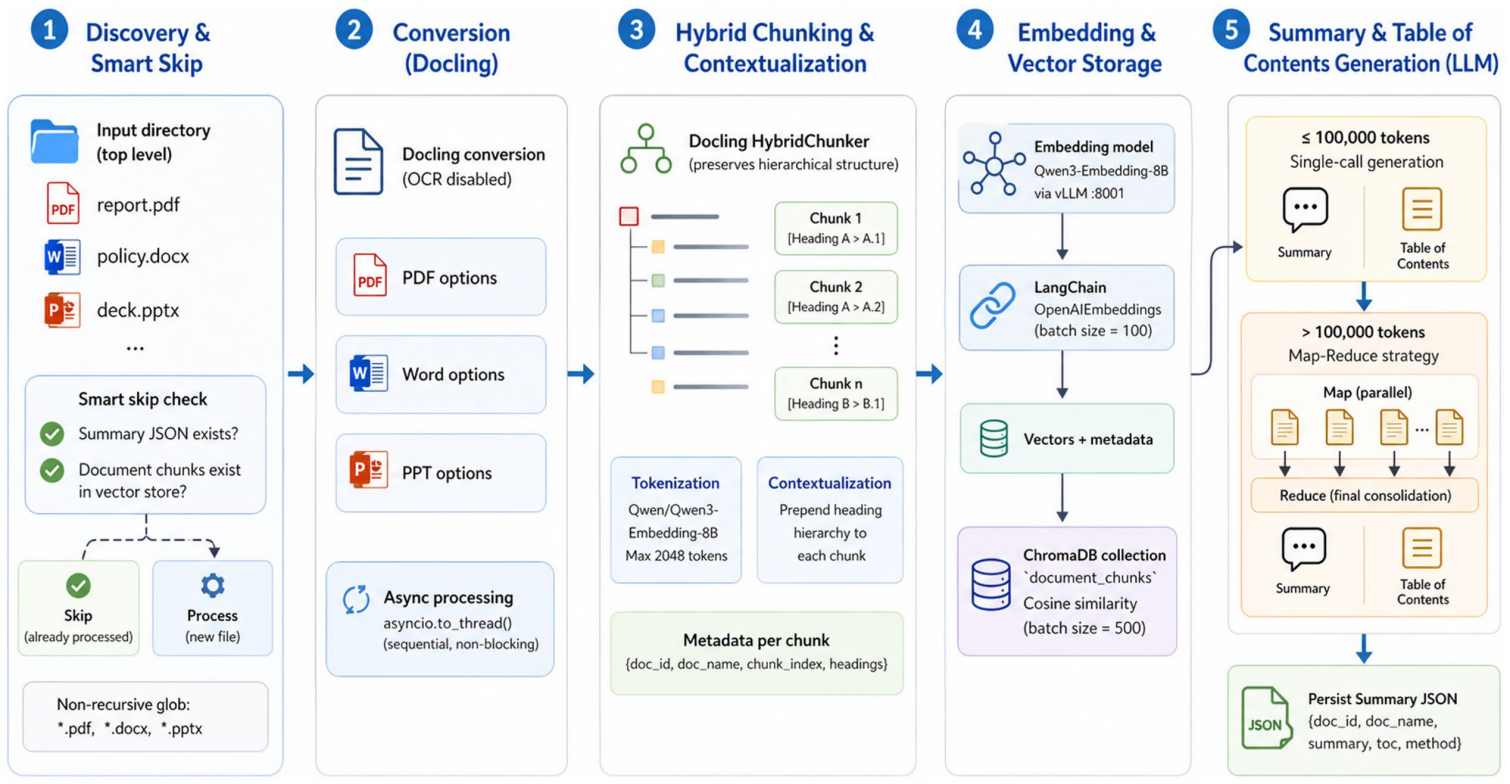
Advanced document ingestion and contextualization pipeline. Comprehensive workflow for automated document conversion and embedding. The process begins with a smart skip check to bypass previously processed files, followed by conversion using Docling. A Docling HybridChunker preserves the document’s hierarchical structure (headings and subheadings) during contextualization. Chunks are embedded using a Qwen3-Embedding-8B model and stored in ChromaDB, while an LLM utilizes map-reduce strategies to generate overarching summaries and tables of contents for large documents.

The complete SCORM 1.2 ZIP archive was successfully uploaded to and launched from the institutional LMS (lms.digitalegiz.kz). Each lesson rendered correctly, displaying structured HTML content with headings, emphasized bold key terms, and learning objectives derived from the source documents (Figure 7). Navigation between lessons functioned as expected via sequential prev/next controls.

**Figure 8:**
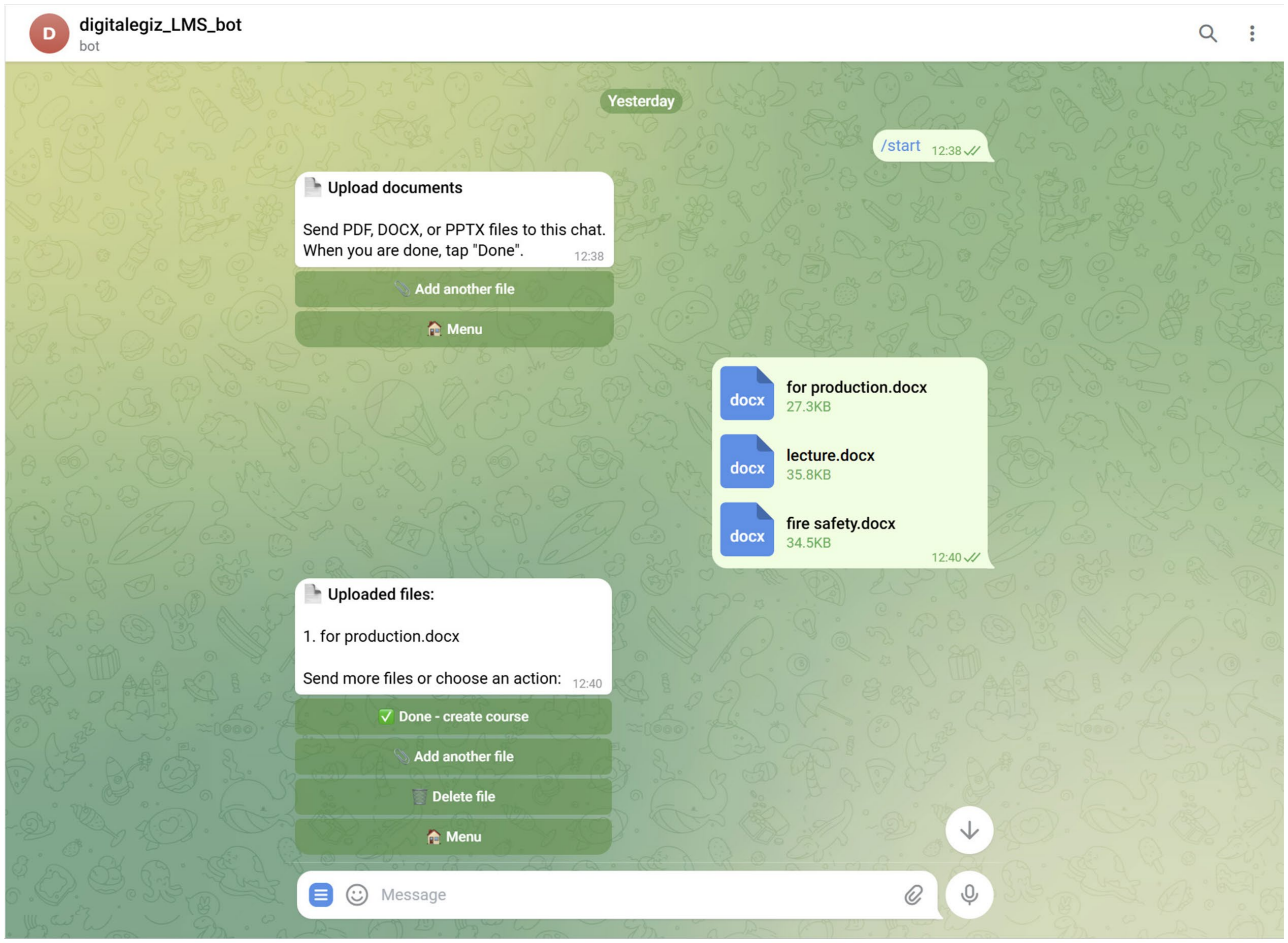
User interface for automated course generation initialization. Screenshot of the digitalegiz_LMS_bot Telegram interface demonstrating the document upload phase. The user interacts with the chatbot to submit various source files (e.g., DOCX documents covering production guidelines and fire safety). The interface utilizes inline keyboard buttons to allow the user to finalize the upload and trigger the backend course creation process.

**Figure 9:**
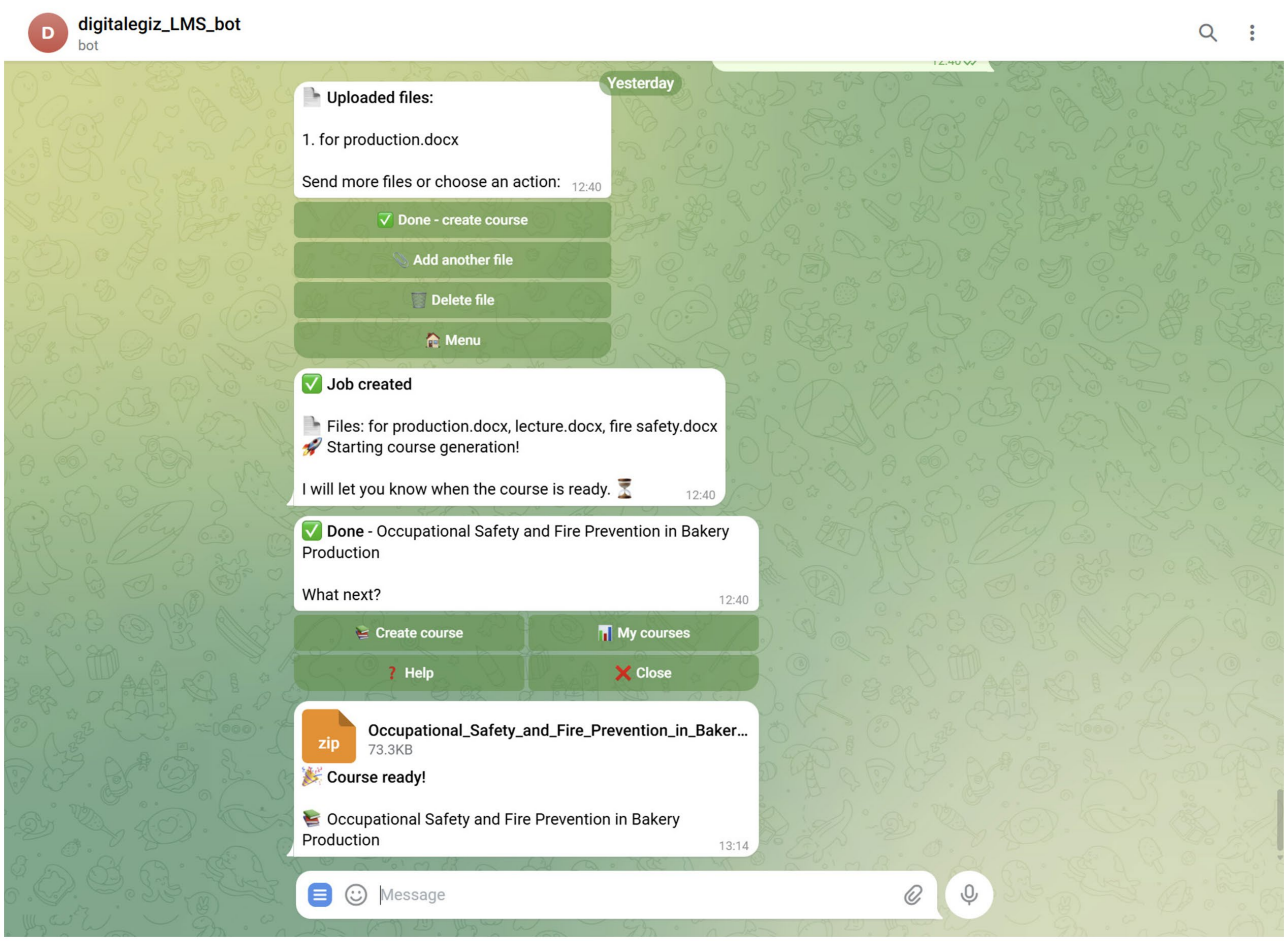
Course delivery and output via conversational agent. Continuation of the digitalegiz_LMS_bot user interaction, depicting the execution and delivery phase. The system acknowledges the job creation, alerts the user that course generation is in progress, and ultimately delivers the compiled SCORM 1.2 course as a downloadable ZIP file titled “Occupational Safety and Fire Prevention in Bakery Production”.

Each of the 16 lessons included an auto-generated Knowledge Check assessment comprising multiple-choice questions, true/false statements, and a matching exercise (Figures 8, 9). All assessment types rendered with correct interactive controls: radio buttons for MCQs, binary true/false buttons, and dropdown-based matching with shuffled right-column options. Assessment items were semantically grounded in lesson content—for example, matching exercises paired production roles—such as, baker, equipment operator, and sanitation worker—with their specific safety requirements drawn directly from source documents.

### 3.3 Retrieval evaluation dataset construction

To evaluate retrieval independently from downstream lesson generation, we constructed a query–passage evaluation set from a separate corpus of four Kazakhstani labor legislation documents. The construction procedure followed the general retrieval evaluation logic used in prior RAG evaluation work, where domain queries are paired with reference passages and retrieval quality is measured using ranking metrics such as Precision@k, Recall@k, NDCG@k, and MRR (Ghosh and Mittal, 2025).

**Figure 10:**
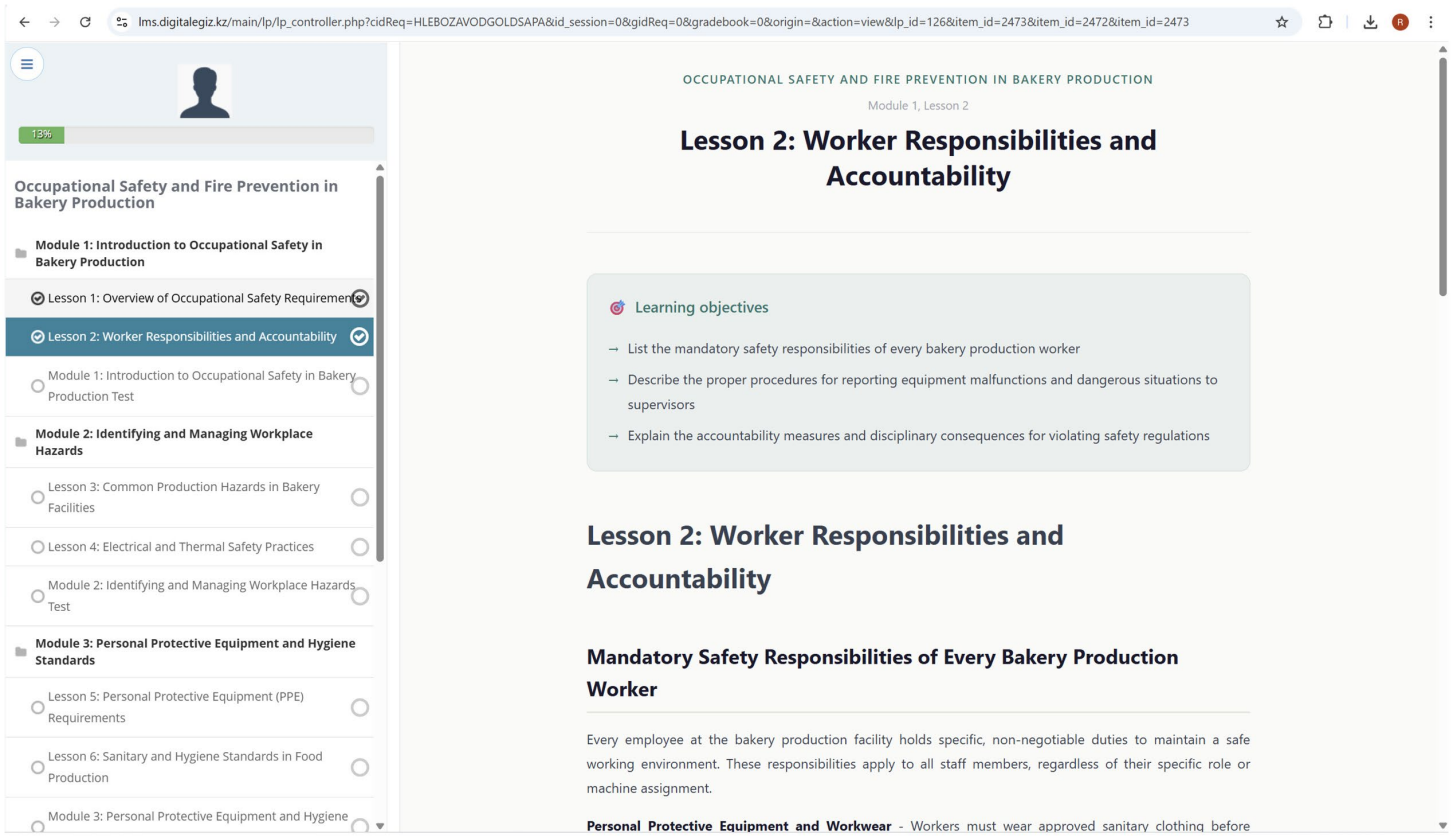
Deployed course interface within a learning management system. A representative view of the automatically generated course running natively within an LMS environment. The interface demonstrates the accurate deployment of the SCORM package, featuring a hierarchical module and lesson navigation menu on the left pane, and the rendered content for “Lesson 2: Worker Responsibilities and Accountability”—including targeted learning objectives—on the main display area.

Evaluation queries were generated with an LLM from semantically chunked source passages. For each selected passage, the prompt asked the model to generate one natural, user-style search query that a real person might use to find the information in the passage. The prompt instructed the model to avoid trivial heading repetition, avoid copying source phrases verbatim, ensure that each query was answerable from the given chunk, and classify each query by difficulty (easy, medium, or hard) and category (definition, procedure, comparison, or factual). This classification strategy was used to encourage diversity across factual, procedural, definition-oriented, and comparison-style queries. The resulting evaluation set contained 231 LLM-generated queries (Figure 10).

**Figure 11:**
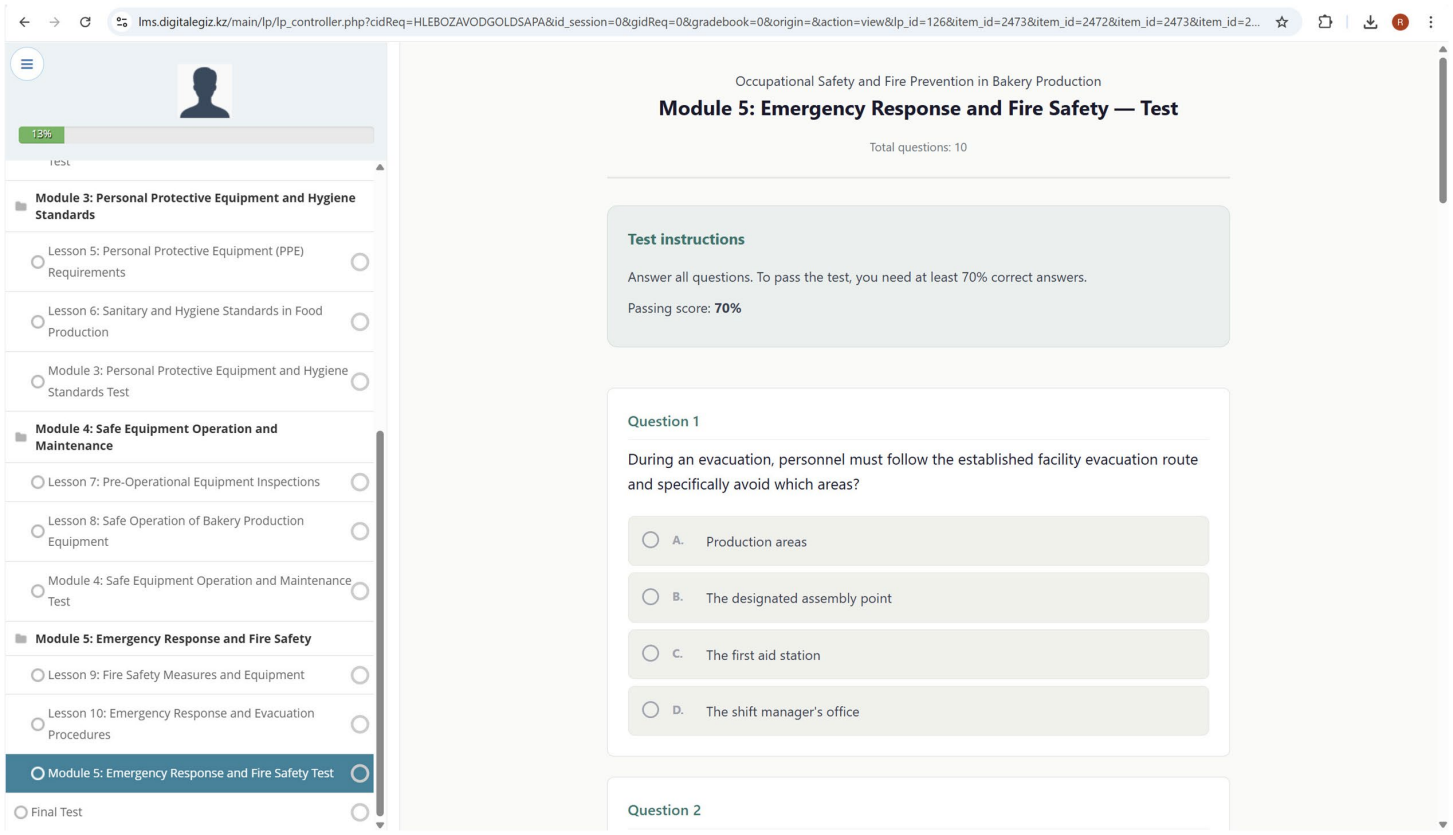
Automated assessment deployment: multiple-choice interface. A screenshot of the deployed Learning Management System (LMS) interface illustrating an automatically generated module assessment. The view displays a standard multiple-choice question format within the “Emergency Response and Fire Safety” module. The interface includes dynamic test instructions, passing score thresholds (set to 70%), and the hierarchical course navigation menu on the left, demonstrating the seamless integration of AI-generated SCORM 1.2 quiz components.

Ground-truth relevance was defined at the passage level. The source passage used to generate each query was marked as the ground-truth relevant passage, resulting in one relevant passage per query. Dataset validation verified whether required fields were present, query identifiers were unique, relevant passage identifiers existed in the ChromaDB collection, and difficulty and category labels used the expected values. This design isolates the ability of the retriever to recover the source passage from which each query was generated (Figure 11).

### 3.4 Retrieval evaluation on the labor legislation corpus

To evaluate the retrieval component independently from content generation quality, we conducted a dedicated intrinsic evaluation of the dense retrieval pipeline on a separate regulatory corpus comprising four Kazakhstani labor legislation documents, comprising 1,965 semantically segmented passages. Retrieval quality was measured using standard IR metrics—Precision@k, Recall@k, NDCG@k, and mean reciprocal rank (MRR)—across 231 LLM-generated evaluation queries. Two retrieval configurations were compared. The corpus-wide configuration searches all chunks without document-level restriction, replicating the condition faced by the Architect agent when the source document for a given query is unknown. The document-scoped configuration restricts retrieval to the single document containing the relevant passages, representing the upper-bound performance achievable when document identity is known in advance. Under corpus-wide retrieval, the pipeline achieved Precision@5 = 0.217, Recall@15 = 0.503, NDCG@15 = 0.369, and MRR = 0.388. Document-scoped retrieval substantially improved all metrics, reaching Recall@15 = 0.817 and MRR = 0.684. The full comparison is presented in Table 1.

**Table 1:** Retrieval metrics under corpus-wide and document-scoped configurations.

Metric	Corpus-wide	Document-scoped	Δ
Precision@5	0.217	0.381	+0.164
Recall@15	0.503	0.817	+0.314
NDCG@15	0.369	0.691	+0.322
MRR	0.388	0.684	+0.296

This table presents a performance comparison between two retrieval strategies—Corpus-Wide and Document-Scoped—evaluated across standard Information Retrieval (IR) metrics. The “$\Delta$” column indicates the absolute improvement gained by transitioning to a scoped retrieval approach.

**Figure 12:**
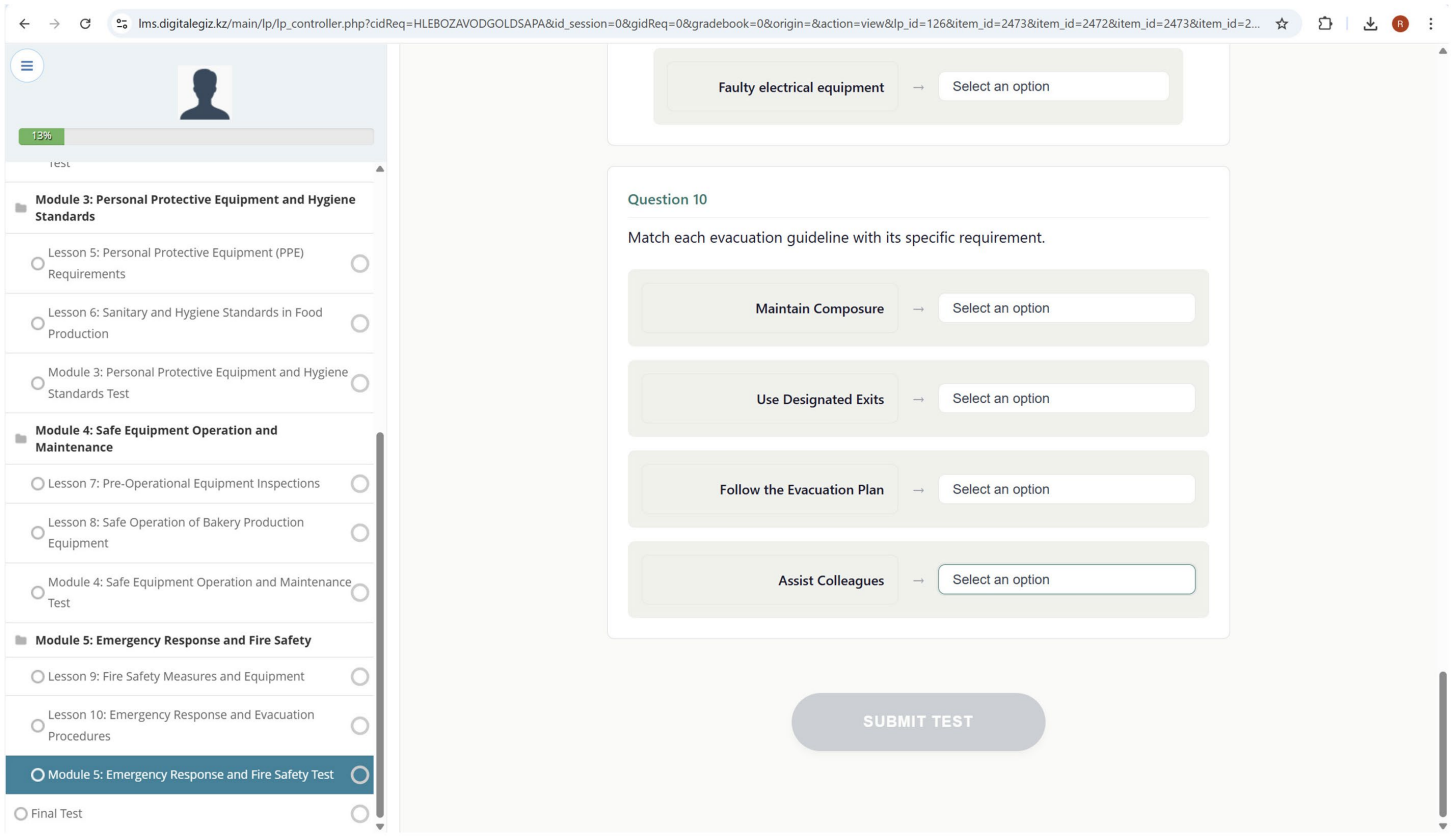
Automated assessment deployment: interactive matching interface. A continuation of the deployed LMS assessment interface, highlighting the system’s capability to generate diverse evaluation formats. This view features a complex matching-style question where learners must pair evacuation guidelines (e.g., “Maintain Composure” or “Use Designated Exits”) with their specific requirements using dropdown menus. The “Submit Test” button at the bottom illustrates the functional conclusion of the module’s interactive assessment, completely generated via the underlying LLM pipeline and native SCORM packaging.

The 0.314 gap in Recall@15 between configurations quantifies the cost of cross-document disambiguation—the primary retrieval challenge in enterprise corpora where regulatory terminology overlaps across documents. An error analysis identified two dominant failure modes: queries about specific topics retrieving thematically adjacent but contextually incorrect passages from other documents and narrow-scope queries failing to match tabular content whose formatting diverges from natural language query phrasing (Figure 12).

These results have two direct implications for the content generation pipeline. First, the multi-query strategy—generating three to five diverse search queries per lesson rather than a single query—partially compensates for the moderate per-query recall, as independent queries cover different facets of the lesson topic and their union substantially increases overall passage coverage. Second, the neural reranking stage with Qwen3-Reranker-8B addresses the precision deficit inherent in bi-encoder retrieval. By rescoring the deduplicated candidate pool with a cross-encoder that captures fine-grained relevance signals, the re-ranker elevates the most contextually appropriate passages into the top-20 context window provided to the content generation model. The retrieval evaluation reported in this study was conducted without the re-ranker active; adding reranking is expected to improve effective precision, which is consistent with established findings in the retrieval-augmented generation literature.

## 4 Discussion

The results demonstrate that a multi-agent retrieval-augmented pipeline can fully automate the transformation of proprietary enterprise documents into deployment-ready e-learning courses without manual authoring intervention. Applied to a real-world occupational safety corpus from a manufacturing enterprise, the system produced a coherent 4-module, 16-lesson SCORM course—complete with structured content and auto-validated assessments—within 16 min of document upload. This end-to-end automation addresses a gap that existing educational AI systems have left largely unaddressed: the generation of training content grounded exclusively in proprietary organizational knowledge.

The autonomous behavior of the Architect agent warrants particular attention. Without any user-specified learning goals, the agent decomposed three heterogeneous documents into a thematically coherent modular structure that directly reflected the source material—covering general safety provisions, equipment operation, fire emergency procedures, and sanitation requirements as distinct modules. This emergent decomposition suggests that the combination of document summarization, table-of-contents extraction, and semantic search provides sufficient signal for the agent to identify natural thematic boundaries in an enterprise corpus. This stands in contrast to systems such as SARA AI (Althaf et al., 2025) and ACG (Anwar et al., 2025), which require explicit keyword input or user-defined topic lists to initiate course generation, placing the structural design burden on the user rather than the system.

The RAG-grounding architecture addresses a fundamental risk in enterprise training automation: content hallucination. General-purpose language models lack access to proprietary organizational knowledge by definition, and generating training material from parametric memory alone risks producing inaccurate or non-compliant content—an unacceptable outcome in regulated environments where safety procedures carry legal weight. By constraining content generation exclusively to retrieved source chunks, the proposed system is designed such that generated content remains grounded in retrieved source passages, thereby making each generated passage traceable, in principle, to the original documents. However, empirical verification of this traceability through human expert review remains a direction for future work, unlike systems such as AutoLMS (Abbasi et al., 2024) and the MotorIA system (Rodriguez-Hernández et al., 2020), which either operate on public knowledge sources or do not provide explicit grounding guarantees, our system ensures content generation is exclusively constrained to retrieved source chunks. In the evaluated corpus, this grounding was particularly consequential, as occupational safety instructions contain precise procedural requirements—such as specific protective equipment, exact reporting chains, and defined emergency actions—where paraphrasing from general knowledge would introduce unacceptable imprecision.

From a deployment perspective, the Telegram bot interface eliminates the need for command-line expertise, enabling HR and training managers to initiate course generation directly from mobile devices by uploading documents to a familiar messaging interface. The 16-min end-to-end latency represents the measured end-to-end processing time for the evaluated corpus, and the resulting SCORM 1.2 archive requires no custom integration—it uploads directly to any standards-compliant LMS.

Several limitations of the current evaluation must be acknowledged. First, the evaluated GOLD SAPA corpus is intentionally small, approximately 1,000 words across three documents; therefore, it represents a proof-of-concept demonstration rather than a large-scale production evaluation. Although this corpus is realistic for a small manufacturing onboarding scenario, it does not establish performance on multi-hundred-page manuals, large policy repositories, or document collections with extensive topical overlap. Second, the study validates technical feasibility, SCORM compatibility, retrieval behavior, and LMS rendering; however, it does not include a human-centric learner study. Consequently, the effect of the generated course on learning outcomes, knowledge retention, learner satisfaction, or onboarding performance remains unmeasured. Future research should include controlled studies with real onboarding cohorts and comparisons against manually authored training materials. Third, the scalability of the multi-agent orchestration has not yet been evaluated on significantly larger repositories. The current pipeline coordinates ingestion, architecture generation, lesson generation, assessment generation, validation, and packaging, with lesson-level generation parallelized after course structure approval. For larger repositories, bottlenecks may arise in document routing, cross-document retrieval, re-ranker input limits, GPU memory, and concurrent LLM calls. Production-scale deployment would require hierarchical retrieval, metadata-aware routing, queue-based execution, caching of summaries and embeddings, monitoring of agent failures or retries, and resource-aware scheduling across LLM, embedding, reranking, and vector-store services.

This study does not include a human-subject evaluation. While the system generates instructional content intended for learners, no empirical measurements of comprehension, knowledge retention, learner satisfaction, or behavioral outcomes are reported. Consequently, claims regarding learning effectiveness or onboarding impact are outside the scope of this study. The evaluation is strictly limited to system functionality, retrieval performance, and successful deployment in an LMS environment.

### 4.1 Evaluation scope

This study evaluates (1) end-to-end pipeline execution and generation latency; (2) retrieval performance using standard IR metrics (Precision@k, Recall@k, NDCG@k, and MRR); (3) structural validity of the generated course, including module decomposition and assessment format correctness; and (4) SCORM 1.2 compliance and successful deployment in an institutional LMS. However, this study does not evaluate learner comprehension, knowledge retention, user satisfaction, or onboarding effectiveness. These dimensions represent essential directions for future human-subjects research.

An additional practical constraint concerns infrastructure requirements. The pipeline depends on three concurrently served neural models—QuantTrio/Qwen3.5-122B-A10B-AWQ (the main generative LLM, served via a self-hosted vLLM 0.17.2rc1 on a two-node NVIDIA GB10 cluster), Qwen/Qwen3-Embedding-8B (embedding model), and Qwen/Qwen3-Reranker-8B (reranker)—which collectively require substantial GPU resources. This hardware dependency may represent a barrier for small organizations without access to dedicated inference infrastructure, although the emergence of cloud-based vLLM hosting services progressively reduces this constraint. Full deployment specifications are reported in Supplementary material S1.

Future studies should pursue evaluation across larger and more diverse enterprise corpora spanning multiple domains, languages, and document formats. Automated content quality assessment—using reference-free metrics or LLM-as-a-judge evaluation—would reduce reliance on manual expert review and enable systematic comparison of architectural variants. Controlled human-subject studies measuring comprehension, knowledge retention, and learner satisfaction with generated courses would establish whether technical fidelity translates into measurable training effectiveness.

## Data Availability

Publicly available datasets were analyzed in this study. This data can be found at: https://github.com/aleka07/scorm_agents.
