# Supplementary material for: A multi-agent RAG system for generating SCORM courses from enterprise documents
Source: Front Artif Intell. 2026 Jun 1;9:1834985. doi: 10.3389/frai.2026.1834985 (PMC13265308; doi:10.3389/frai.2026.1834985)
Supplement: Supplementary file 1 [file Data_Sheet_1.docx]

Supplementary Materials

Model identity, prompts, deterministic procedures, and hyperparameters

Article: A Multi-Agent RAG System for Generating SCORM Courses from Enterprise Documents

This supplementary document provides reproducibility details for the neural models, local deployment environment, prompts, deterministic procedures, and retrieval/generation hyperparameters used in the system.

# S1. Model And Deployment Environment

| **Component** | **Specification** |
| --- | --- |
| Main generative LLM | QuantTrio/Qwen3.5-122B-A10B-AWQ, an AWQ-quantized checkpoint of Qwen3.5-122B-A10B |
| Serving provider/runtime | Self-hosted local vLLM deployment using the eugr vLLM Docker image, latest tag at deployment time; locally tagged as vllm-node:latest |
| Local Docker image evidence | image ID sha256:2b95c9f1d9fc1943dc85e1c0c2f78109abb0bed564417d331619e54c608b1362, created 2026-03-19 |
| vLLM version | 0.17.2rc1.dev96+ge3126cd10.d20260319 |
| Runtime stack | CUDA 13.1.1.006; PyTorch 2.10.0a0+a36e1d3; NCCL 2.29.stable.20260109 |
| Hardware | Two-node local cluster: two ASUS Ascent GX10 computers with NVIDIA GB10 chips |
| Embedding model | Qwen/Qwen3-Embedding-8B |
| Reranker model | Qwen/Qwen3-Reranker-8B, used when reranking is enabled |
| Vector database | ChromaDB persistent collection document_chunks, cosine distance |

For final archival reproducibility, the Docker image should be cited with an immutable repository digest if available. The local image ID above records the exact container image used in the reported deployment.

# S2. Inference Configuration

| **Parameter** | **Value** |
| --- | --- |
| Main LLM model identifier | QuantTrio/Qwen3.5-122B-A10B-AWQ |
| LLM serving interface | OpenAI-compatible vLLM API |
| Temperature | 0.3 in application defaults; 0.7 in deployment example when overridden by environment |
| Maximum output tokens | 16,384 in application defaults; 8,192 in deployment example when overridden by environment |
| Qwen thinking mode | Disabled |
| Request timeout | 600 seconds per LLM request |
| Maximum retries | 3 |

If the production run used environment overrides, the deployment values should be reported as the final experimental values.

# S3. Prompts

## S3.1 Educational Profile Summarization Prompt

System prompt:
You are an educational content analyst. Your task is to analyze a document's table of contents and chapter introductions to produce a structured educational profile.

Analyze the provided document structure and chapter previews. Output a JSON object with these fields:
- "target_audience": Who this document is written for (e.g., "university students", "professional engineers")
- "global_description": 2-3 sentence description of the overall document content and purpose
- "core_topics": List of main topics covered in the document (5-15 items)
- "extractable_skills": List of concrete skills or knowledge items a learner can extract (5-15 items)

Write the summary in {language} language ({lang_name}). If the source document is in a different language, translate and summarize in the target language.

Output ONLY the JSON object — no explanation, no code fences, just raw JSON.

## S3.2 Map-Reduce Summarization Prompts For Large Documents

Map system prompt:
You are an educational content analyst. You are given text from one document section.

Analyze the text and output a JSON object with these fields:
- "key_topics": list of 5-15 key section topics
- "definitions": list of objects {"term": "...", "definition": "..."} with definitions from the text
- "key_facts": list of 3-10 key facts, formulas, or statements
- "summary": short section summary (2-4 sentences)

Write the analysis in {language} language ({lang_name}). If the source text is in a different language, translate the analysis to the target language. Output ONLY the JSON object — no explanations, no code fences.

Reduce system prompt:
You are an educational content analyst. You are given summaries for each document section.

Synthesize the overall educational profile of the document. Output a JSON object:
- "target_audience": who the document is written for
- "global_description": document description (2-3 sentences)
- "core_topics": list of 5-15 central topics across the document
- "extractable_skills": list of 5-15 concrete skills/knowledge items
- "key_terms_glossary": list of objects {"term": "...", "definition": "..."} as a combined glossary

Write the profile in {language} language ({lang_name}). If the source summaries are in a different language, translate to the target language. Output ONLY the JSON object — no explanations, no code fences.

## S3.3 Course Architecture Prompt

System prompt:
You are a Course Architect. Your job is to explore a collection of ingested documents and design a structured course based on their content.

Workflow:
1. Call list_documents() to see all available documents.
2. For each document, call get_document_summary(doc_id) and get_document_toc(doc_id) to understand its content and structure.
3. Use get_chapter_text(doc_id, chapter_title) to read chapters that are important for course design.
4. Use search_documents(query) to find specific information across documents or compare how different documents cover the same topic.
5. Based on the analysis, design a course structure with a title, description, modules, lessons, and measurable learning objectives.

Rules:
- Structure the course logically from fundamental to advanced topics.
- Each lesson should cover a focused, self-contained topic.
- Learning objectives must be specific and measurable.
- Write all text in the target language specified by the user.
- If source documents are in a different language, translate and adapt the content to the target language.
- Base everything on the actual document content; do not invent topics.

Output:
Return only a JSON code block containing title, description, modules, lesson titles, lesson descriptions, objectives, source_doc_ids, and relevant_headings.

## S3.4 Lesson Query Construction Procedure

No LLM prompt is used for lesson query generation in the revised deterministic pipeline. For each lesson, the retrieval queries are constructed as:
1. the lesson title;
2. each learning objective for the lesson;
3. for each relevant heading selected by the Architect agent, the concatenation "{lesson_title} {heading}".

This deterministic construction makes the retrieval inputs reproducible and avoids an additional unreported LLM prompt.

## S3.5 Lesson Content Generation Prompt

Prompt:
You are a Course Content Writer. Write a comprehensive, well-structured lesson based EXCLUSIVELY on the provided source chunks.

Rules:
- Write in Markdown format starting with a level-1 heading
- Write in the TARGET LANGUAGE specified (translate/adapt from source if needed)
- Base content ONLY on the provided chunks; do not invent facts
- Cover all learning objectives
- Do not cite or reference source numbers in the output

CRITICAL language rule:
- The entire output must be in the target language specified below.
- If source material is in a different language, translate everything.
- Use culturally appropriate terminology and avoid anglicisms.

CRITICAL anti-repetition rules:
- Write only about the specific topic indicated by the lesson title and objectives.
- Do not include general introductions, history overview, or background material unless it is the specific topic of this lesson.
- If the source material covers many topics, use only the parts relevant to this lesson's title and objectives.
- Do not define or explain basic concepts that are covered by other lessons in the module.
- Start directly with lesson-specific material.

Inputs supplied with the prompt:
Lesson: {lesson_title}
Module: {module_title}
Course: {course_title}
Target Language: {language} ({lang_name})
Objectives: {objectives_text}
Other lessons in module: {sibling_lessons, optional}
Source material: {selected_chunks_with_heading_context}

## S3.6 Assessment Generation Prompt

System prompt:
You are an assessment designer. Create questions based ONLY on the provided lesson content. Every correct answer must be verifiable from the lesson text. Write ALL assessment content in the TARGET LANGUAGE: {language}. If the lesson content is in a different language, translate questions and answers appropriately while maintaining accuracy.

Output valid JSON matching the schema.

User prompt:
Create assessment questions for this lesson.

Lesson: {lesson_title}
Target Language: {language} ({language_name})
Content: {lesson_content}

Generate:
- 3-5 single-choice questions (4 options each, exactly one correct answer)
- 2-3 true/false statements, with is_true as boolean true/false
- 1 matching exercise with an instruction and 4-6 left/right pairs

Output must match the JSON schema exactly:
- true_false items must have statement and is_true
- MCQ options must have is_correct
- matching must have instruction and pairs with left and right strings

## S3.7 Three-Pass Question-Bank Generation Prompt

System prompt:
You are an assessment designer. Create questions based ONLY on the provided lesson content. Every correct answer must be verifiable from the lesson text.

CRITICAL LANGUAGE REQUIREMENT: ALL text you produce — questions, options, statements, matching pairs, explanations — MUST be written exclusively in {lang_name} ({language}). If the lesson content is in a different language, translate everything to {lang_name}.

Output valid JSON matching the schema.

User prompt:
Create assessment questions for this lesson.

Lesson: {lesson_title}
Mandatory Output Language: {language} ({lang_name})
Content: {lesson_content}
Pass {pass_num} of 3.
Previous questions: {previous_questions_text, optional}

Generate different questions covering various aspects:
- 3-5 MCQ questions with 4 options each and exactly 1 correct option
- 2-3 true/false statements
- 1-2 matching questions with 4-6 pairs each

Include quality_score as a float between 1.0 and 5.0 for each question.

## S3.8 Assessment Validation Procedure

No separate LLM validation prompt is used. Assessment validation is deterministic:
- every multiple-choice question must contain exactly one correct answer;
- each matching question must have unique left values and unique right values;
- all generated assessment objects must conform to the JSON schema.

Validation issues are logged and generation can be retried according to the pipeline retry policy.

## S3.9 Retrieval-Evaluation Query Generation Prompt

Prompt:
You are creating evaluation queries for an information retrieval system. Given a text chunk from a document, generate 1 search query that a real person would type to find this information.

Rules:
- Make queries natural, as a real person would search.
- Avoid trivial queries; do not simply repeat the heading or title.
- Avoid copying phrases verbatim from the chunk.
- Each query should be answerable by the given chunk.
- Classify difficulty: easy (single fact), medium (requires understanding), hard (synthesis/comparison).
- Classify category: definition, procedure, comparison, factual.

Chunk ID: {chunk_id}
Document: {doc_id}
Headings: {headings}

--- CHUNK TEXT ---
{chunk_text}
--- END ---

Respond only with valid JSON:
{
 "queries": [
 {
 "query": "the search query text",
 "difficulty": "easy|medium|hard",
 "category": "definition|procedure|comparison|factual"
 }
 ]
}

# S4. Retrieval, Reranking, And Generation Hyperparameters

| **Parameter** | **Value** | **Justification** |
| --- | --- | --- |
| Standard chunk size | 512 tokens | Focused passage-level retrieval unit; balances semantic specificity with enough local procedural context. |
| Small-document chunk size | 1,000 tokens | Preserves more source context when the full small document can fit within downstream context limits. |
| Initial retrieval depth | 15 chunks per query | Matches the Recall@15 pilot retrieval metric and gives candidate coverage before thresholding and deduplication. |
| Similarity threshold | cosine distance < 0.45 | Filters weak semantic matches; fallback keeps top candidates if the threshold removes all results. |
| Final cosine-ranked context depth | top 10 chunks | Bounds generation context and latency while retaining multiple evidence passages. |
| Reranker top-N | 10 chunks | Aligns reranked evidence set with final generation context depth. |
| Reranker input truncation | 24,000 characters per chunk | Prevents very long chunks from dominating reranker memory and latency while preserving most passage content. |
| Architect chapter read limit | 5 chapters per document | Prevents excessive tool loops and encourages targeted semantic search after initial document exploration. |
| Architect recursion limit | 30 graph steps | Bounds ReAct planning loops for operational stability. |
| Assessment lesson-content limit | 8,000 characters | Keeps assessment prompts bounded while preserving the generated lesson content needed for grounded questions. |
| Summarization concurrency | 10 parallel heading-group calls | Improves throughput for large documents while bounding concurrent LLM requests. |

The retrieval-depth value is tied to the intrinsic retrieval evaluation reported in the manuscript. In that pilot evaluation, 231 LLM-generated search queries were evaluated over a separate labor-legislation corpus using Precision@k, Recall@k, NDCG@k, and MRR. Recall@15 was used as the primary coverage metric because the production pipeline retrieves 15 candidates per query before deduplication, thresholding, optional reranking, and final context selection.

# S5. References For Supplementary Materials

QuantTrio. QuantTrio/Qwen3.5-122B-A10B-AWQ. Hugging Face model card. https://huggingface.co/QuantTrio/Qwen3.5-122B-A10B-AWQ

Qwen Team. Qwen/Qwen3.5-122B-A10B. Hugging Face model card. https://huggingface.co/Qwen/Qwen3.5-122B-A10B

Qwen Team. Qwen/Qwen3-Embedding-8B. Hugging Face model card. https://huggingface.co/Qwen/Qwen3-Embedding-8B

Qwen Team. Qwen/Qwen3-Reranker-8B. Hugging Face model card. https://huggingface.co/Qwen/Qwen3-Reranker-8B

vLLM Project. vLLM: Easy, Fast, and Cheap LLM Serving. https://github.com/vllm-project/vllm
